# Application of the Ottawa Charter Five Priority Areas of Action for Public Health to an Institution-Wide Diabetes Care Promotion

**DOI:** 10.3390/ijerph18041543

**Published:** 2021-02-05

**Authors:** Min-Hua Lin, She-Yu Chiu, Wen-Chao Ho, Hui-Ying Huang

**Affiliations:** 1 Department of Nutrition, China Medical University, Taichung 40402, Taiwan; minhua5356@gmail.com (M.-H.L.); emily572005@gmail.com (H.-Y.H.); 2 Department of Dietetics, Yunlin Christian Hospital, No. 375, Shichang S. Rd.Xiluo Township, Yunlin 64866, Taiwan; 3 Institute of Population Health Sciences, National Health Research Institutes, No. 35, Keyan Road, Zhunan Town, Miaoli County 35053, Taiwan; koca345678@gmail.com; 4 College of Public Health, China Medical University, No. 91, Hsueh-Shih Road, Taichung 40402, Taiwan; 5 Department of Nursing & Graduate Institute of Nursing, Asia University, No. 500, Lioufeng Road, Wufeng, Taichung 41354, Taiwan

**Keywords:** Ottawa Charter, health promotion, diabetes mellitus care, health behavior, qualitative study

## Abstract

This study was the first institution-wide health promotion program in Taiwan to apply the five priority areas for taking action in public health highlighted in the Ottawa Charter for diabetes patients. We aimed to improve the quality of home care received by diabetic patients by training health care professionals in health promotion. This program consisted of developing personal skills, reorienting health services, strengthening community actions, creating supportive environments, and building healthy public policy. It was applied in the Yunlin Christian Hospital located in central Taiwan from August 2011 to November 2011. A health-promoting education course consisting of weight control, diabetes care, and quality management for diabetes was developed and applied to all 323 hospital staff. Then, hospital staff volunteers and diabetes patients were recruited to participate in the program. A total of 61 staff volunteers and 90 diabetes patients were involved in this study. Staff volunteers were trained to participate in communities to provide care and guidance to patients with diabetes. The World Health Organization Quality of Life(WHOQOL)-BREF-Taiwan Version questionnaires were investigated before and after implementation of this program for the patients. A health-promoting lifestyle profile questionnaire was filled by the staff. The investigation data were then analyzed by statistical methods. The diabetes patients experienced a significant increase in their satisfaction with health and health-related quality of life as well as significant improvements in health-promotion and self-management behaviors (*p* < 0.05). In addition, staff volunteers significantly consumes food from the five major groups than the other staff (*p* < 0.05). Various improvements in health-promoting behaviors were observed amongst the hospital staff and the diabetic patients. Our project could be a reference for other medical organizations to implement an institution-wide health-promotion program for diabetic patients.

## 1. Introduction

According to the 2013 International Diabetes Federation (IDF) Diabetes Atlas (6th edition, Brussels, Belgium), diabetes has become a global concern [1]. In 2011, there were an estimated 366 million diabetic individuals worldwide. The number of diabetic patients is predicted to reach 642 million by 2040 [2]. The predominance of type II diabetes among seniors raises public health concerns. Diabetes was the fifth major cause of death in Taiwan in 2016 (standardized mortality of 25.4 per 100,000 people). The Yunlin Christian Hospital is located in Xiluo Township, Yunlin County, Taiwan. In 2016, diabetes was the fifth major cause of death in Yunlin County. The standardized mortality of diabetes was 23 per 100,000 people in Yunlin County and 33.1 per 100,000 people in Xiluo Township. In Taiwan, Yunlin county has the second highest ratio of seniors. Various factors such as level of education, literacy, and living alone can affect the self-care ability of diabetic patients. The poor control of blood sugar in patients with diabetes is correlated with macrovascular disease, loss of sight, kidney failure, neurological lesions, and amputation [3,4,5,6,7]. Macrovascular complications are the main causes of death in diabetes patients [8].

Previous retrospective research has revealed that an enhanced treatment for diabetes after discharge may reduce the risk of re-admission for patients with poor control of blood sugar [9,10]. The successful management of diabetes is not only dependent upon medical treatment, but also includes diabetes self-management such as self-care actions, a balanced diet, physical activity, weight control, and blood sugar self-monitoring.

Various randomized controlled studies worldwide have supported the effect of lifestyle changes in preventing or delaying type II diabetes [11,12,13,14]. Some studies have indicated psychological issues, especially depression, that could affect the will to follow treatment and worsen the prognosis [15,16,17,18,19,20]. Additionally, scholars claim that the use of only metabolic indicators in evaluating the effects of health education may paint an incomplete picture, and the results obtained may be biased [21,22,23,24]. In addition to a careful experimental design, a life quality questionnaire should be applied to comprehensively evaluate the subjective feeling of the patients toward physical, psychological, and social factors as well as their surrounding environment [25,26]. In 1986, the Ottawa Charter for Health Promotion issued by the World Health Organization (WHO) defined health promotion as “the process of enabling people to increase control over and improve their health” [27]. The WHO Ottawa Charter highlights five priority areas for action in public health to facilitate disease prevention and control: building healthy public policy, creating supportive environments, strengthening community action, developing personal skills, and reorienting health care services toward the promotion of health [28,29]. The Charter articulates health in all policies and their frameworks. Therefore, an institution-wide health promotion program based on the five priority areas for taking action that were highlighted in the Ottawa Charter may be useful for diabetic patients. The purpose of this study was to develop and apply this institution-wide program for patients with diabetes in Taiwan and to investigate whether it could improve the quality of home care for these patients. We aimed to improve the quality of home care received by diabetic patients by training health care professionals in health promotion.

## 2. Research Methods

### 2.1. Intervention Background

The studied hospital is a community style health promotion hospital. The vision of this hospital is to be the guardian of the health of the community. The missions of this hospital are to reach excellence, innovation, selfless devotion, and service of humbleness. The objectives of this hospital are to generally improve the health of the community, to provide therapy for all people, to facilitate the staff’s self-fulfillment, and to share the gospels alongside serving people. This program was designed to fulfill the purpose of the studied hospital, which is to serve not only the patients and their family, but also the staff, community, and organization of the hospital. With the involvement and commitment of all staff members, the strategy of organizational development is intended to effectively allocate resources and extend functions. Additionally, the capability of taking action, goal management, communication, and cooperation will be enhanced to make changes in the aspects of the culture, organization, environment, and working procedures of the hospital.

To ensure full support for this institution-wide program, a diabetes health-promotion committee organization chaired by the hospital superintendent was established, and the Department of Dietetics was assigned to provide health education to all hospital staff members and patients with diabetes who were included in the study. Additionally, hospital staff volunteers were recruited and trained to participate in communities to provide care and guidance to patients with diabetes. The goal of the guidance was to promote the family of diabetic patients to develop a long-term and continuous care model that included regular exercise, regular blood sugars self-monitoring, a balanced diet, and maintaining an ideal weight. We aimed to work with the patient’s family as a unit to help strengthen the implementation of a healthy lifestyle for the diabetic patient. In this way, patients can reconstruct healthy behaviors, delay the onset of diabetes complications, and improve their quality of life.

### 2.2. Participant Inclusion

The study protocol was approved by the Institutional Review Board (IRB) in Changhua Christian Hospital (approval number 110903), and we followed the guidelines of the Institutional Review Board (IRB) of Changhua Christian Hospital for experimentation with human subjects. The included subjects were 323 staff members and community diabetes patients in the Yunlin Christian Hospital located in central Taiwan. The staff volunteers were recruited primarily from the staff who had diabetes, an impaired glucose tolerance (IGT), or an impaired fasting glucose (IFG). The inclusion criteria for patients with diabetes were as follows: diagnosed with diabetes ICD-9-250 or type II diabetes by a physician; Yunlin County resident; 20–80 years of age; conscious, capable of communication in Taiwanese or Mandarin; and willingness to participate in the study. The patients were provided with the objectives of the study by trained interviewers.

Furthermore, the patients were informed of their rights regarding study participation. A written consent form was then signed by the patients. The participants were interviewed personally before and after the implementation of the program, respectively, each time for 15 min. The World Health Organization Quality of Life Brief (WHOQOL-BREF)-Taiwan Version questionnaire was filled out [30] and turned in by the staff volunteer. The questionnaire included questions covering four categories including physical conditions, psychological conditions, social relations, and the environment. The patient’s subjective satisfaction level was described by a scale of five sequential scores; higher scores indicated a better life quality.

The hospital staff members were asked to fill a health-promoting lifestyle profile questionnaire before and after the implementation of the program [31]. The questionnaire consisted of two parts: characteristics of demography and health promotion lifestyles that include self-fulfillment (eight items), responsibility for health (eight items), stress handling (nine items), interpersonal support (six items), nutrition (five items), and exercise (four items). In total, 40 items were included in this questionnaire. The questionnaires were given to the staff members by the supervisors of each unit and were turned over to the supervisors in the sealed envelope after being filled out anonymously.

### 2.3. Intervention

The studied hospital implemented a hospital-wide health promotion program for diabetes care based on the five priority areas for taking action in public health highlighted in the Ottawa Charter. The workable details are described in the following subsections.

#### 2.3.1. Action 1: Develop Personal Skills

The studied hospital asked all 323 staff members to complete courses on diabetes care and quality management that were provided on a digital learning platform. The courses included the following: (1) weight control; (2) diabetes care; and (3) quality control for diabetes. Among these courses, the diabetes care course was required for all staff.

The courses of diabetes care for the staff volunteers included the following: (1) 1.5 h on the self-monitoring of blood sugar; (2) 2 h on carbohydrate substitution; (3) 2 h on diet recording; and (4) 1.5 h of fitness exercises. The volunteers received one of the above-described courses and were evaluated by the health educators specialized in diabetes care. Those who passed the evaluation were then provided with a certificate as a volunteer for diabetes care.

The education and training courses provided by the volunteers to the diabetic patients and their family included the following: (1) blood sugar self-monitoring techniques; (2) exercise methods; (3) methods of foot care; (4) diet log maintenance; and (5) recording blood sugar self-monitoring results.

#### 2.3.2. Action 2: Reorient Health Services

The studied hospital organized a “Committee of Institution-wide Health Promotion for Diabetes”. The committee consists of the hospital director and all first-level supervisors. The hospital director asked all the first-level supervisors to promote diabetes prevention with the patients’ family as the basic operation unit and encouraged the staff to actively participate in such development. The committee also established an annual work plan and objectives as well as monthly meetings, promoting the plan among all staff members. The committee also encouraged the staff to become volunteers and step into the communities to provide self-care skills to diabetes patients and their families.

The staff volunteers performed the following: (1) selected three patients in the nearby community for health promotion and established a meeting with the patients and their family; (2) made weekly phone calls to the patient to provide care and supervision on the implementation of blood sugar self-monitoring, exercise, diet log maintenance, and medication; and (3) provided a monthly report to the volunteer team leaders on the challenges and issues faced by the patient’s family. The reports were then summarized by the team leaders into a report prepared for the Committee of Institution-wide Health Promotion for Diabetes.

The studied hospital is a Christian hospital with regulations about voluntary service. The employees are required to serve in the community as volunteers for a minimum of 12 h each year. Volunteers for diabetes patients need to participate in service for 4 h each month in the duration of personal leave. The hospital rewards those who serve as volunteers for more than 20 h a year. The training course for the volunteers to attend to diabetes patients contained the main points as follows:

1. To understand how to improve the health conditions of diabetic patients properly.
2. To understand the importance of selecting nutritious food, exercising regularly, and self-monitoring of blood sugar.
3. To ensure that the volunteers are able to provide health advice and counselling.
4. To help hospitals build/promote health-related events.
5. To make sure volunteers practice what they preach about health improving activities.

The safety measures for the volunteers were as follows:

1. When it is not the volunteer’s family or relatives’ community, three persons serve as a group due to safety concerns.
2. Each volunteer is provided with a whistle that can be brought with them during visits to protect themselves.
3. Teaching the volunteers how to avoid and handle situations of being treated improperly (such as sexual harassment) when serving the opposite gender. Furthermore, the training of raising alerts is highlighted.

#### 2.3.3. Action 3: Strengthen Community Actions

In order to help diabetes patients in the community to establish healthy lifestyle, volunteers for diabetes patients went into the community to serve families with diabetes patients. The volunteers had to do the following:

1. Choose three families of diabetic patients near the volunteers’ home as service objects and establish a health plan with the patients’ family.
2. Volunteers called the patients every week to supervise their self-monitoring of blood sugar level, exercise, diet records, and follow the doctor’s medication instructions.
3. Collect health care reports from the families of diabetic patients every month, report to the captain of volunteers to organize and report to the committee.

The volunteers in this study established a “home visit record” for each patient/family. The record included the following: (1) Basic patient information; (2) biochemical abnormality follow-up evaluation and target setting; (3) health risk factor evaluation (smoking status, alcohol consumption, and chewing of betel nuts); (4) evaluation of self-care behaviors for diabetes (diet control, self-monitoring of blood sugar, exercise); (5) items and plans of health education; (6) evaluation of effects of health education; and (7) signature collection for home visits.

#### 2.3.4. Action 4: Create Supportive Environments

The studied hospital provided the following health promotion environments to the staff. (1) Establishing sports clubs such as a bicycle club and aerobic dance club. The hospital provides the space and the employee welfare committee pays for some of the lecturer fees. (2) Providing staff with knowledge relating to weight loss. (3) Encourage the staff to exercise after work. (4) Discounts on the purchase of sporting equipment. (5) Providing a half day of official leave and reward to encourage the staff to form groups and participate in the sports games held by the hospital. (6) A notebook for the staff to self-monitor and record their method of losing weight.

In order to encourage the community to choose healthy food, the nutritionist helped local restaurants provide free food calorie calculation and nutrition labels. To encourage diabetic patients to record their own self-care history and to design rewards accordingly, the staff volunteers visited the patient and their family every week and discussed the implementation of self-care and the records kept with the patients. The staff needed to check with the family members whether the records were complete. Furthermore, the volunteers provided the following services to the patients: (1) evaluate whether the diet met the principles of high dietary fiber and a low amount of oil; (2) evaluate whether the patients followed the instructions of the nutritionist regarding carbohydrate intake and maintained a complete record of daily meals in a diet log; and (3) whether the status of blood sugar self-monitoring records were kept. Those who met the target were given points and rewarded for the points collected.

#### 2.3.5. Action 5: Build Healthy Public Policy

Trained and qualified volunteers selected and made health plans with three families of diabetic patients as the service objects in the community and assisted the diabetes patients to reach the following goals and behaviors of health and self-care during the program’s duration: (1) for those with a body mass index (BMI) >24, loss of 10% of the weight in six months; (2) for those with a wider waistline, males should achieve a waist circumference <90 cm, and females should achieve a waist circumference <80 cm; (3) the pre-meal blood sugar target was <100 mg/dL for those with a high risk of diabetes and <120 mg/dL for the diabetic patient; (4) for those with hypertension, the target BP was <130/85 mmHg; (5) for those with high blood lipids, the target low-density lipoprotein (LDL) was <100 mg/dL; (6) exercise for >150 min every week; (7) perform blood sugar self-monitoring at least once a week; (8) blood sugar self-monitoring records provided to the physicians; (9) ensure that patients follow the guidance of the physician regarding medication; and (10) follow the plan established by the nutritionist regarding carbohydrate intake, and maintain a complete record of daily meals in their diet log.

### 2.4. Statistical Analysis Methods

In total, 93 patients satisfied the inclusion criteria, and 90 were included in the study. The SPSS 18.0 software (SPSS Inc., Chicago, IL, USA) was utilized for statistical analysis, and the data was analyzed for percentage, mean, and standard deviation. Additionally, the data were subjected to the independent sample t-test and univariate analysis of variance.

## 3. Results

Characteristics of the diabetes health care volunteers are presented in Table 1. A total of 90 patients with diabetes were recruited, and their characteristics are presented in Table 2.

### 3.1. Results of Patients

After the implementation of the institution-wide health promotion program in the studied hospital, we obtained the following results:

After the care and guidance given by the volunteers to diabetic patients and their family, the patients were provided with the WHOQOL-BREF. In total, an 81 and 77 response rate were collected before and after the implementation of the plan, respectively. The results indicated the following: (1) A significant (p < 0.05) positive increase in the satisfaction regarding health and health-related quality of life, which were evaluated on a scale of 10 scores (Table 3); (2) regarding the physical and biochemical indicators of the cared individuals before and after the implementation of the program, body weight (BW), percent of ideal body weight (%IBW), body mass index (BMI), Ante Cibum glucose (Sugar AC), Post Cibum glucose (Sugar PC), hemoglobin A1c (HbA1C), systolic blood pressure (BP Systolic), and diastolic blood pressure (BP Diastolic) were tested using the paired t test where significant improvement was found (Table 4); and (3) significant improvements in health-promoting behaviors including blood sugar self-monitoring and following plans given by the nutritionist regarding carbohydrate intake and regular exercise were noted (Table 5). These data were collected from patient records, therefore 90 data were collected.

### 3.2. Results of Hospital Staff and Staff Volunteers

An anonymous questionnaire on health-promoting behaviors was administered to the hospital staff. In total, 323 questionnaires were collected before and after plan implementation. The results revealed significant improvements (*p < 0.05*) in eating a fixed amount at a fixed time, avoiding artificial additives in food, performing guided exercise, pulse measurement during exercise, performing stretching exercises at least three times per week, performing 20–30 min of exercise for a minimum of three times a week, participating in individual health care courses, identifying sources of stress, undergoing cholesterol tests, and attaching importance to the sense of accomplishment. Among the hospital staff who volunteered, 55.3% often consumed food from the five major food groups, which was greater than the 44.1% among those who did not volunteer (*p* < 0.001).

## 4. Discussion

This study presented the results of the implementation of the first hospital-wide diabetes health-promotion program in Taiwan. After the volunteers were trained, they provided diabetes education to the diabetic patients and their families and collected biochemical indicators from the diabetic patients. Although the patients did not show significant improvements in their physical and biochemical indicators in this study, they did show significant improvements in health-promoting and diabetes self-management behaviors including blood glucose self-monitoring, following the dietitian’s plan regarding carbohydrate intake, and engaging in regular exercise.

Obesity not only increases the risk of type 2 diabetes, but also complicates its management. Studies have indicated that specific alterations in human gut microbiota lead to obesity and obesity-related metabolic diseases. Obesity is conditioned by multiple factors, of which microbiota are certainly influential. In a previous study, microbiota and their composition, functions, and ecology were influenced by numerous factors that might be modified by behavior, physical activity, sedentary lifestyle, and the surrounding environment [32]. Above all, eating habits produced profound changes in intestinal flora by changing their composition, ecology, and functionality [33]. A healthy and balanced diet with different molecular mechanisms, some of which directly involve microbiota, has positive health effects [34]. In short, microbiota are certainly linked to human health and disease.

In the present study, 82% of the volunteers were married. These married volunteers had more practice and experience with home care and had less fear in communicating with patients with diabetes and their families. In addition, the married volunteers had more knowledge on diabetes prevention. Therefore, married volunteers contributed substantially to the promotion of self-care actions among patients with diabetes.

Education regarding self-management has been shown to prevent the development of concomitant complications, which may help improve health outcomes [20]. According to national and international guidelines, the overall goal of diabetes treatment should be the prevention of acute and chronic complications while maintaining the patient’s quality of life [21]. Policymakers, therefore, have begun to realize that quality of life is the ultimate goal of all health interventions and is an important and measurable result in determining the outcomes of diabetes care and burden [21,22]. In our study, the average age of the included diabetic patients was 64.1 years. The poor quality of life was found to be related to older age, and the deterioration of the quality of life of the elderly population might be due to an increase in their weakness [23]. This study found that the proposed program did have a significant improvement in the satisfaction regarding the health and health-related life quality of diabetes patients.

A study from Turkey found education to be positively related to physical health, mental health, social relationships, the environment, and overall quality of life [24]. A collaborative and integrated medical team comprising clinical pharmacists and all healthcare professionals and the active support of patients with diabetes could play a vital role in improving patient quality of life [25]. Several studies have reported that this multidisciplinary approach and patient education as well as drug consultation by clinical pharmacists in diabetic care programs lead to improvements in blood glucose control, quality of life, and other clinical outcomes in patients with diabetes [26]. Our research in Taiwan also demonstrated that an institution-wide health promotion program is useful for improving the life quality of diabetes patients.

In this study, significant improvements in physical and biochemical indicators were not found. This might be due to the limited duration of the study, which was only three months. A longer project and assessment of behavior changes covering more than one year is suggested for future research.

## 5. Conclusions

The patients with diabetes and their family served by the hospital volunteers expressed significantly increased satisfaction with their health and health-related quality of life. They exhibited significant improvements in health-promotion and self-management behaviors including blood glucose self-monitoring, adherence to dietitian plans for carbohydrate consumption, and routine exercise. In addition, the hospital staff who were volunteers selected a more balanced diet of the five major food groups than those who were not volunteers.

The model of our project can be a reference for other medical organizations implementing institution-wide health-promotion programs for patients with diabetes. We also suggest that interventions for patients with diabetes and their family should last longer than half a year to potentially obtain more promising results. In addition, the Taiwanese health system requires a comprehensive, long-term master plan centered on the principles of the Ottawa Charter to improve general health and overcome type 2 diabetes in particular.

## Figures and Tables

**Table 1:** Characteristics of the diabetic healthcare volunteers.

Variable		N	%
Gender	Male	15	24.6
	Female	46	75.4
Age (years)	20–29	16	26.2
	30–39	24	61.0
	40–49	20	32.8
	50–59	1	1.6
Supervisor	Yes	21	34.4
	No	40	65.6
Marital	Married	50	82.0
Status	Single	11	18.0
Diabetes high risk	Yes	18	29.5
	No	43	70.5
Professional	Ancillary Staff	21	34.4
	Nurse	21	34.4
	Physiotherapist	7	11.5
	Dietitian	5	8.2
	Pharmacist	3	4.9
	Radiologist	2	3.3
	Senior Technician	2	3.3

**Table 2:** Characteristics of diabetic patients.

Variable		N	%
Gender	Male	48	53.3
	Female	42	46.7
Age	20–44	4	4.1
	45–64	36	40.0
	65–74	31	34.4
	75–100	19	21.1
Education level	University	9	10.1
	High school	14	15.7
	Middle school	12	13.5
	Elementary school	27	30.3
	Illiterate	27	30.3
Language	dialect	43	47.8
	Chinese	47	52.2
Occupation	Retired	29	32.2
	Farmer	29	32.2
	Workers	4	4.4
	Business	10	11.1
	Medicine & bio-tech	2	2.2
	Government official	2	2.2
	Leisure & service	2	2.2
	Housework	7	7.8
	Others	5	5.6
Marital status	Married	88	97.8
	Single	2	2.2
Complications	Neuropathy (yes)	72	82.2
	Neuropathy (no)	7	7.8
	Neuropathy (unknown)	9	10.0
	Nephropathy	62	70.5
	Nephropathy(no)	5	5.6
	Nephropathy (unknown)	21	23.9

**Table 3:** Health promotion lifestyle satisfaction of diabetic patients.

	Pre/Post	N	Mean	Standard Deviation	*p* Value
Health satisfaction	Pre test	81	2.86	0.81	0.038
	Post test	77	3.14	0.85	
Health-related quality of life satisfaction	Pre test	81	3.37	0.80	0.026
	Post test	78	3.44	0.75	

**Table 4:** Differences in the physical and biochemical measurements of diabetic patients before and after the health promotion program.

Item	N	Mean (Paired T)	SD	*p* Value
BW	89	0.0562	0.7762	0.4965
%IBW	88	−0.1080	1.7705	0.5688
BMI	89	−0.0213	0.3961	0.6124
SUGAR AC	78	3.4615	27.3703	0.2675
SUGAR PC	15	−4.6667	28.1645	0.5314
HbA1C	44	0.1136	1.0064	0.4579
BP (Systolic)	37	0.2432	15.6159	0.9250
BP (Diastolic)	37	1.4324	12.1713	0.4787

**Table 5:** Self-care actions before and after the health promotion program.

Variable		Before	After	Fisher	*p* Value
Follows a diabetic diet	Yes	42	48	54.9131	<0.001 *
	No	42	42		
Exercise behavior	Regular	54	60	8.4000	0.0101 *
	Irregular	36	30		
Self-care via glucose monitoring	Yes	40	42	77.3545	<0.0001 *
	No	50	48		

note: * *p* < 0.01.

## Data Availability

The datasets analyzed during the current study are available from the corresponding author on reasonable request.
